# Supplementary material for: Coordination of biradial-to-radial symmetry and tissue polarity by HD-ZIP II proteins
Source: Nat Commun. 2021 Jul 14;12:4321. doi: 10.1038/s41467-021-24550-6 (PMC8280177; doi:10.1038/s41467-021-24550-6)
Supplement: Supplementary file 1 — Supplementary Information [file 41467_2021_24550_MOESM1_ESM.pdf]

Supplementary Figure 1

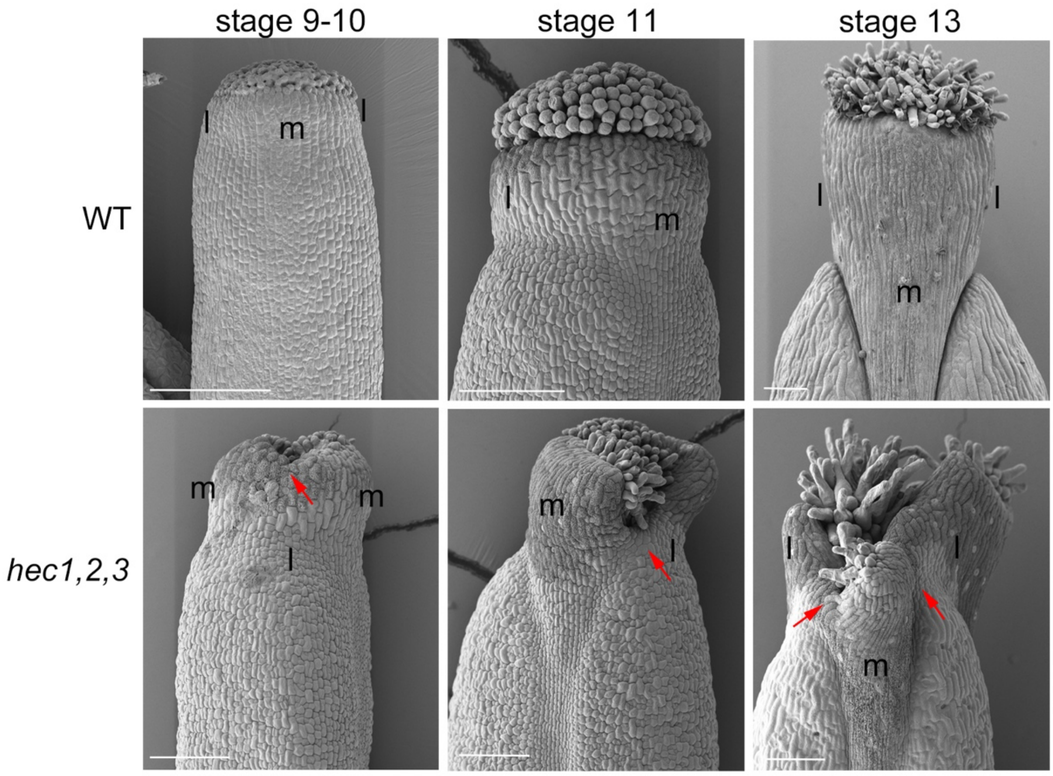

Supplementary Figure 1. *hec1,2,3* mutant style shows a diagonal split phenotype similar to *hat3 athb4*. SEM images of Col-0 wild-type (top panels) and *hec1,2,3* triple mutant (bottom panels) at stage 9-10 (left panels), stage 11 (midle panels), and stage 13 (right panels). Red arrows indicate the position of the split style in the mutant background; m and l represent medial and lateral domains. Scale bars represent 100 µm. Similar results were obtained from two independent experiments.

Supplementary Figure 2

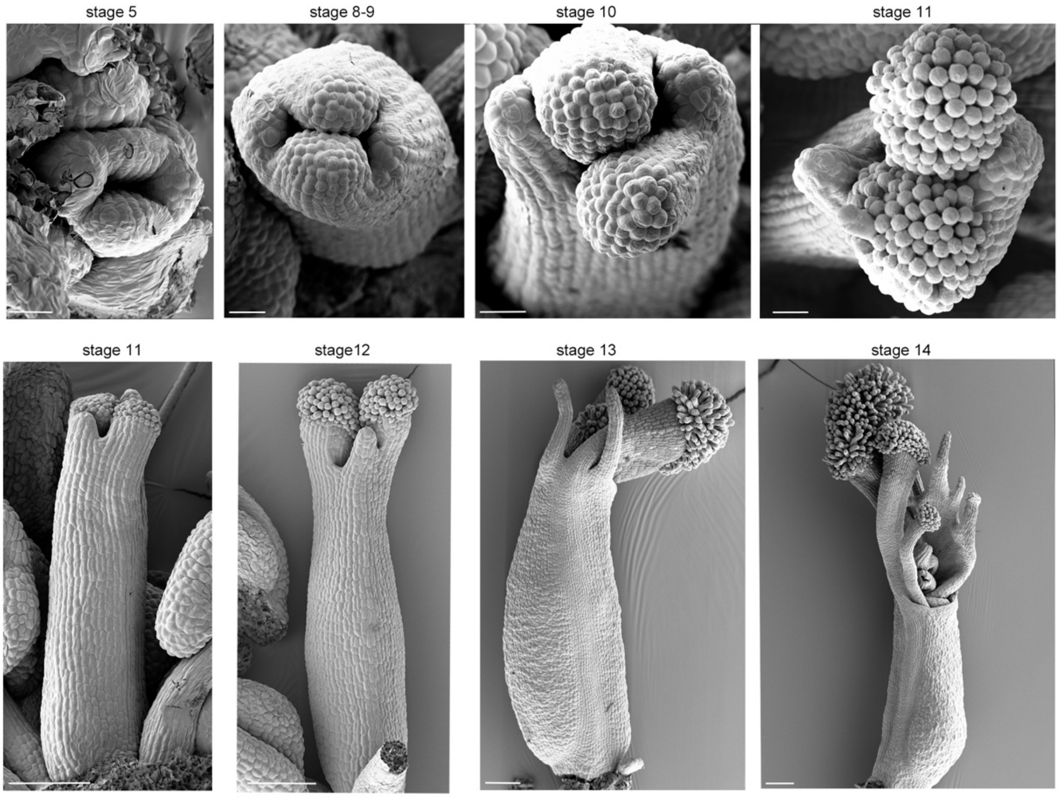

Supplementary Figure 2. *hat3 athb4* gynoecium defects throughout developmental steges. SEM images of *hat3 athb4* gynoecia viewed from the top (top panels) and from the side (bottom panels) at developmental stages depicted above each panel. Scale bars represent 20 µm (top) and 100 µm (bottom). Similar results were obtained from four independent experiments.

Supplementary Figure 3

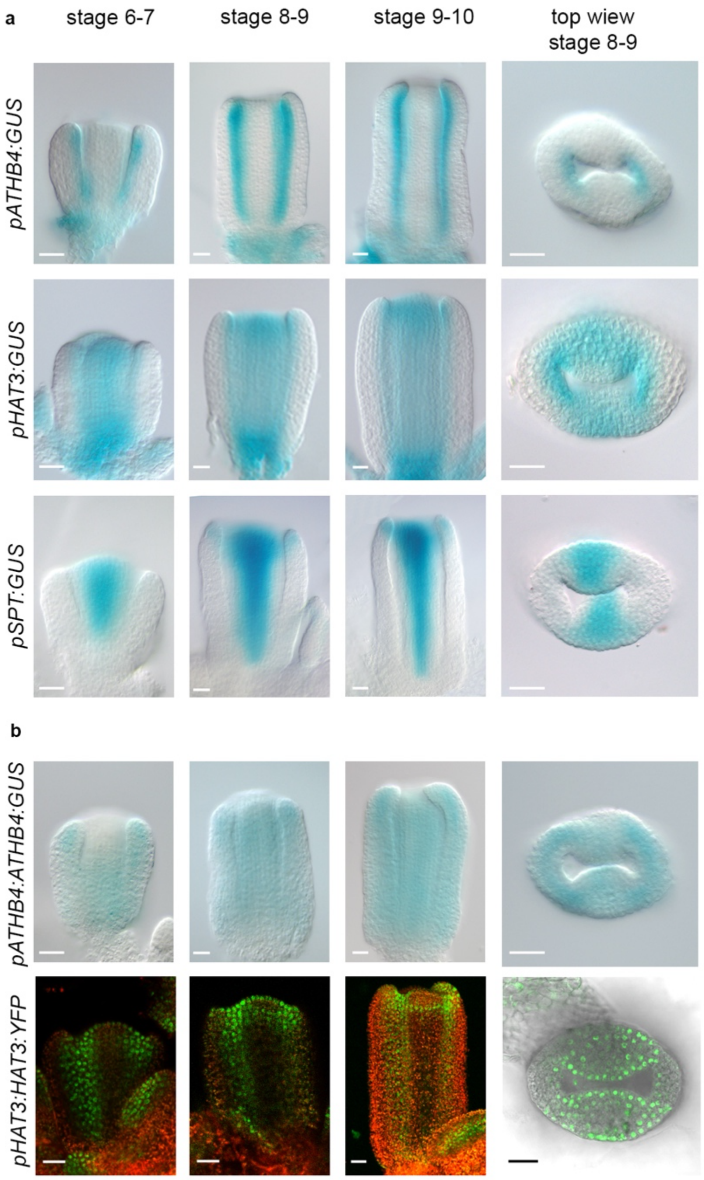

Supplementary Figure 3. *HAT3* and *ATHB4* expression during gynoecium development. a, Optical images of GUS-stained gynoecia of *pATHB4:GUS* (top panels), *pHAT3:GUS* (middle panels), and *pSPT:GUS* (bottom panels) transcriptional fusion lines at different stages of gynoecium development (stages depicted in figure). Similar results were obtained from four independent experiments. **b**, Optical images of GUS-stained gynoecia of the *pATHB4:ATHB4:GUS* translational fusion line (top panels) and confocal images of the *HAT3::HAT3:YFP* translational fusion line (bottom panel) at different stages of gynoecium development. Scale bars represent 20 µm. Similar results were obtained from three independent experiments for *pATHB4:ATHB4:GUS* and from two independent experiments for *pHAT3:HAT3:YFP*.

Supplementary Figure 4

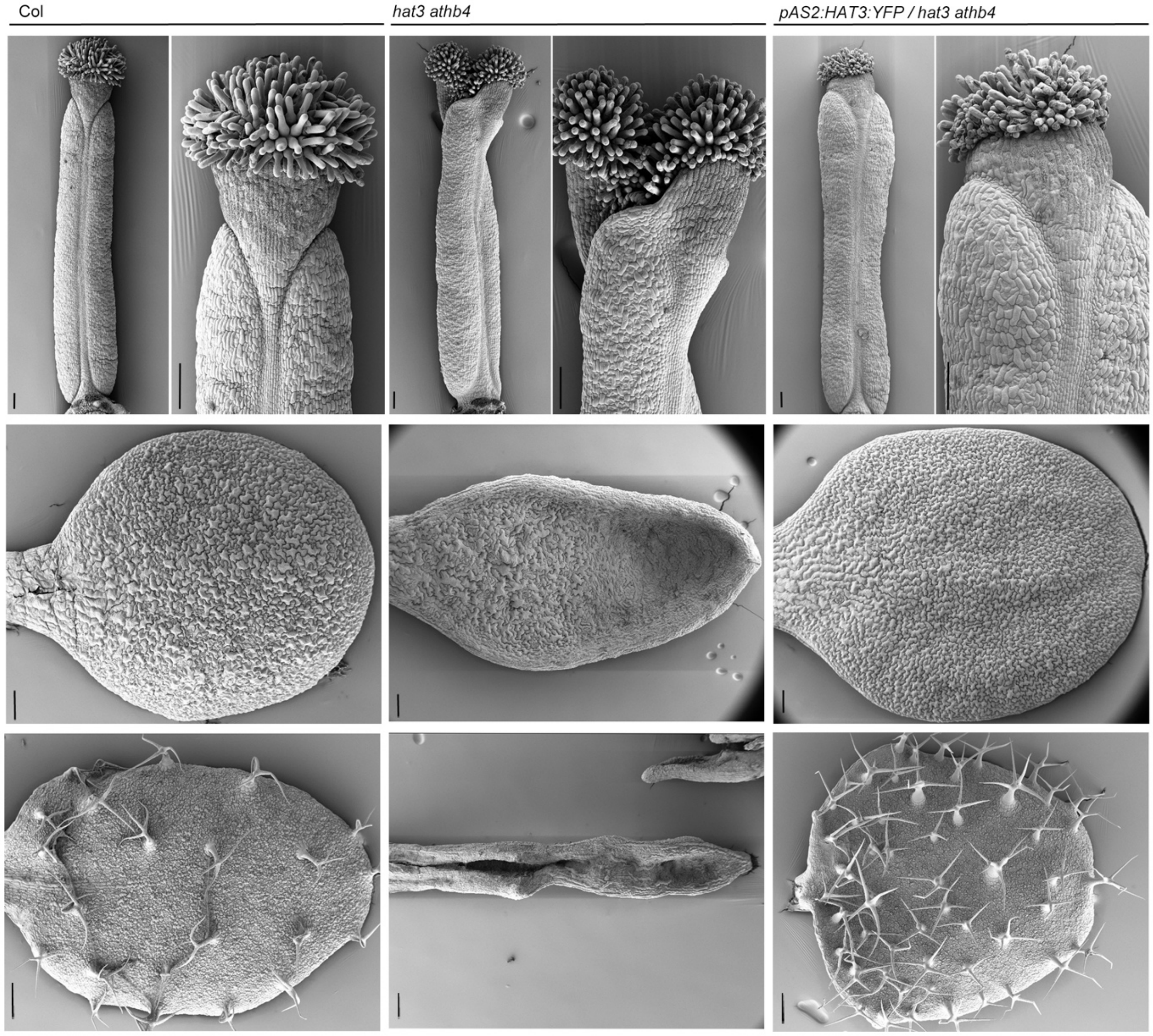

Supplementary Figure 4. Adaxial expression of *HAT3* is sufficient to rescue the *hat3 athb4* gynoecium and leaf patterning defects. SEM images of Col-0 (left), *hat3 athb4* (center), and *pAS2:HAT3:YFP/hat3 athb4* complementation line (right) showing gynoecia and styles at stage 13 (top panels; scale bars represent 100 µm) cotyledons (middle panels; scale bars represent 200 µm) and true leaves (bottom panels; scale bars represent 200 µm). Similar results were obtained from three independent experiments.

Supplementary Figure 5

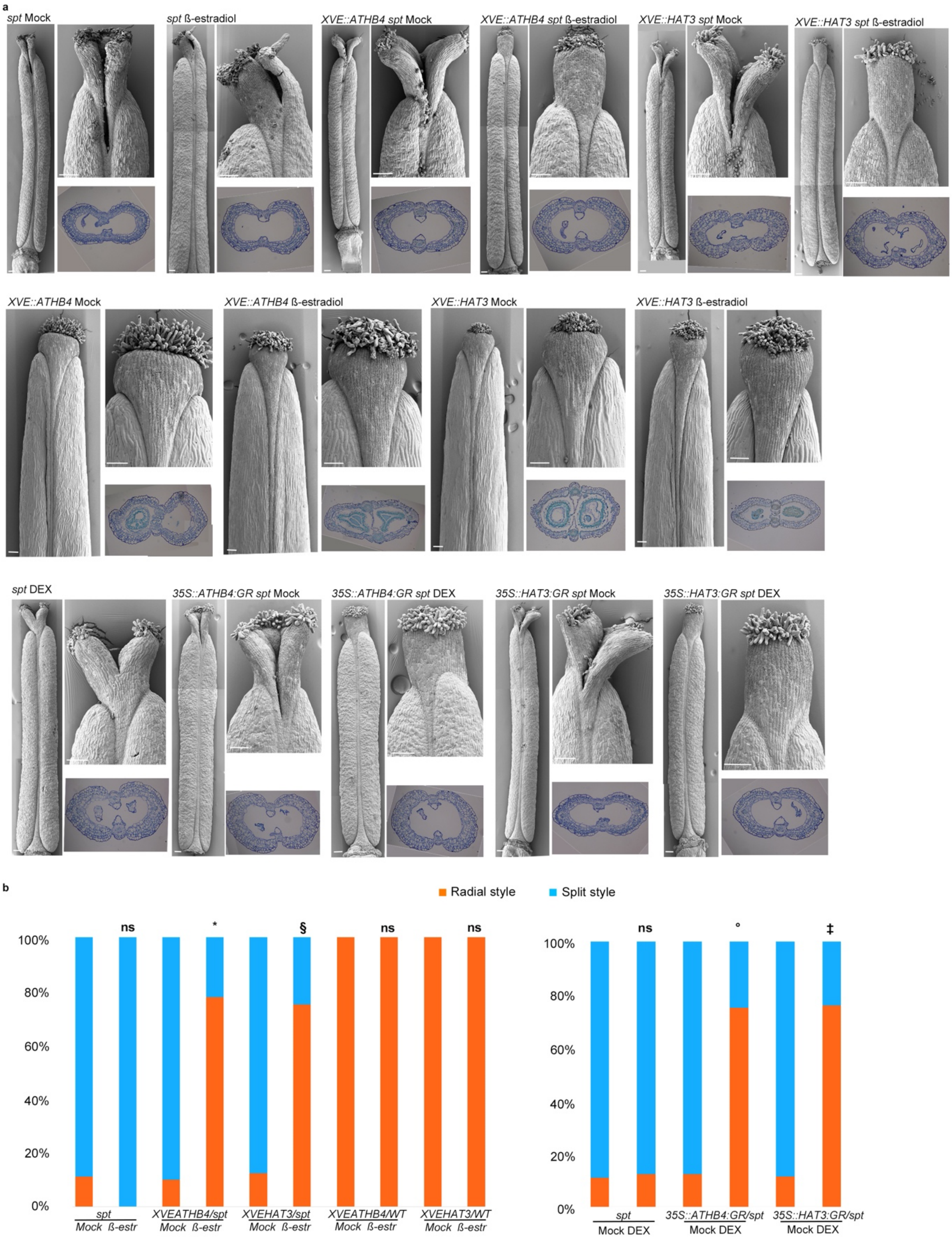

Supplementary Figure 5. Overexpression of *HAT3* and *ATHB4* specifically rescue symmetry defect of the *spt* style but not the transmitting tract. a, SEM images of gynoecia and styles, and toluidine blue-stained ovary cross-sections of overexpression lines of *HAT3* (both *XVE::HAT3* and *35S::HAT3:GR*) and *ATHB4* (both *XVE::ATHB4* and *35S::ATHB4:GR*) in wild-type or *spt* background, treated with either 20 µM ß-estradiol or 10 µM DEX, compared to Mock treated samples, as depicted on individual panels. Scale bars represent 100 µm. **b,** Quantification of the radial (orange bars) and split (blue bars) style phenotype of *HAT3* and *ATHB4* overexpression lines *XVE::HAT3* and *XVE::ATHB4* in wild-type and *spt* background treated with Mock and ß-estradiol (left); and of *35S::HAT3:GR* and *35S::ATHB4:GR* lines in *spt* background treated with Mock and DEX (right). Phenotypic classes were compared using 2 x 2 contingency tables followed by Pearson's chi-square test. Two-tailed P values are as follows: *spt-12* β-estradiol vs *spt-12* mock P=0.014301 (ns; no statistically significant difference); *spt-12 XVE::ATHB4* β-estradiol vs *spt-12 XVE::ATHB4* mock P=0.000013 (*); *spt-12 XVE::HAT3* β-estradiol vs *spt-12 XVE::HAT3* mock P<0.00001 (§); *XVE::ATHB4* β-estradiol vs *XVE::ATHB4* mock P=0.621863 (ns); *XVE::HAT3* β-estradiol vs *XVE::HAT3* mock P=1 (ns); *spt-12* DEX vs *spt-12* mock P=0.790127 (ns); *spt-12 35S::ATHB4:GR* DEX vs *spt-12 35S::ATHB4:GR* mock P<0.00001 (°); *spt-12 35S::HAT3:GR* DEX vs *spt-12 35S::HAT3:GR* mock P<0.00001 (‡). P values <.01 were considered as extremely statistically significant.

Supplementary Figure 6

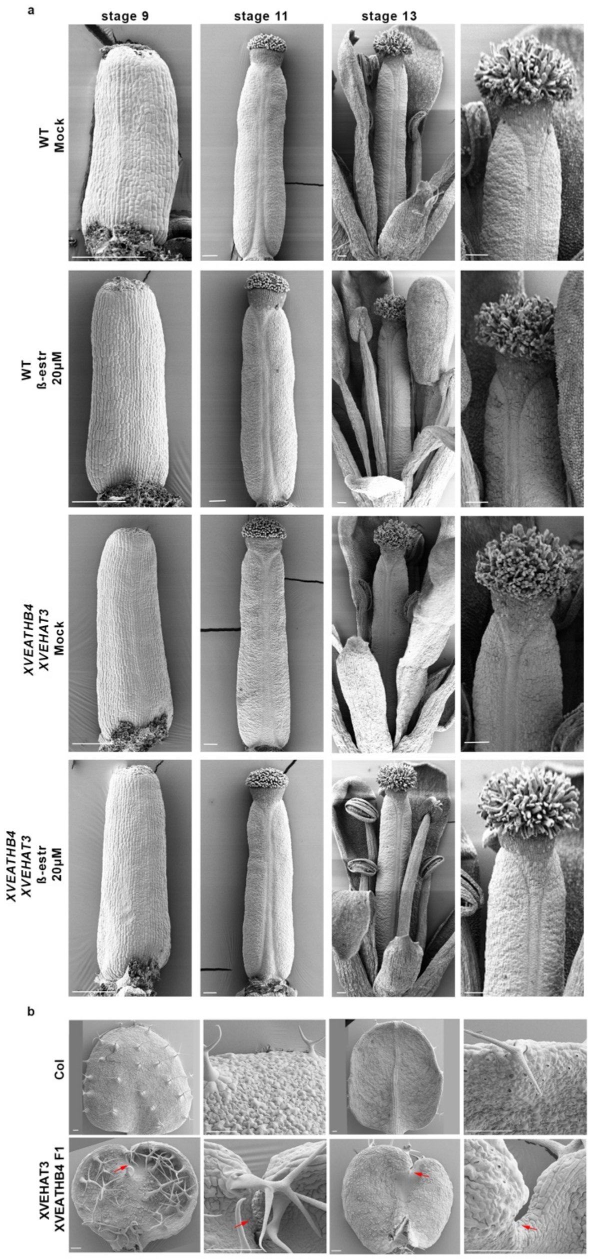

Supplementary Figure 6. Overexpression of *HAT3* and *ATHB4* impacts on leaf development but does not alter gynoecium symmetry. a, SEM images of Col-0 wild-type and *XVE::HAT3* x *XVE::ATHB4* F1 gynoecia at stage 9, stage 11, and stage 13 of their development, treated with 20 µm of ß-estradiol or mock. Scale bars represent 100 µm. **b**, Co-overexpression of HAT3 and ATHB4 enhance bilateral symmetry in leaves. SEM images of first true leaves of seedlings grown on media supplemented with 20 µM ß-estradiol of Col-0 (top) and *XVE::HAT3* x *XVE::ATHB4* F1 (bottom). Adaxial (left) and abaxial (right) sides are shown of the entire leaf surfaces and magnification of their distal bifurcated tips. Red arrows indicate the position of the split leaves. Scale bars indicate 100µm. **a,b** Similar results were obtained from two independent experiments.

Supplementary Figure 7

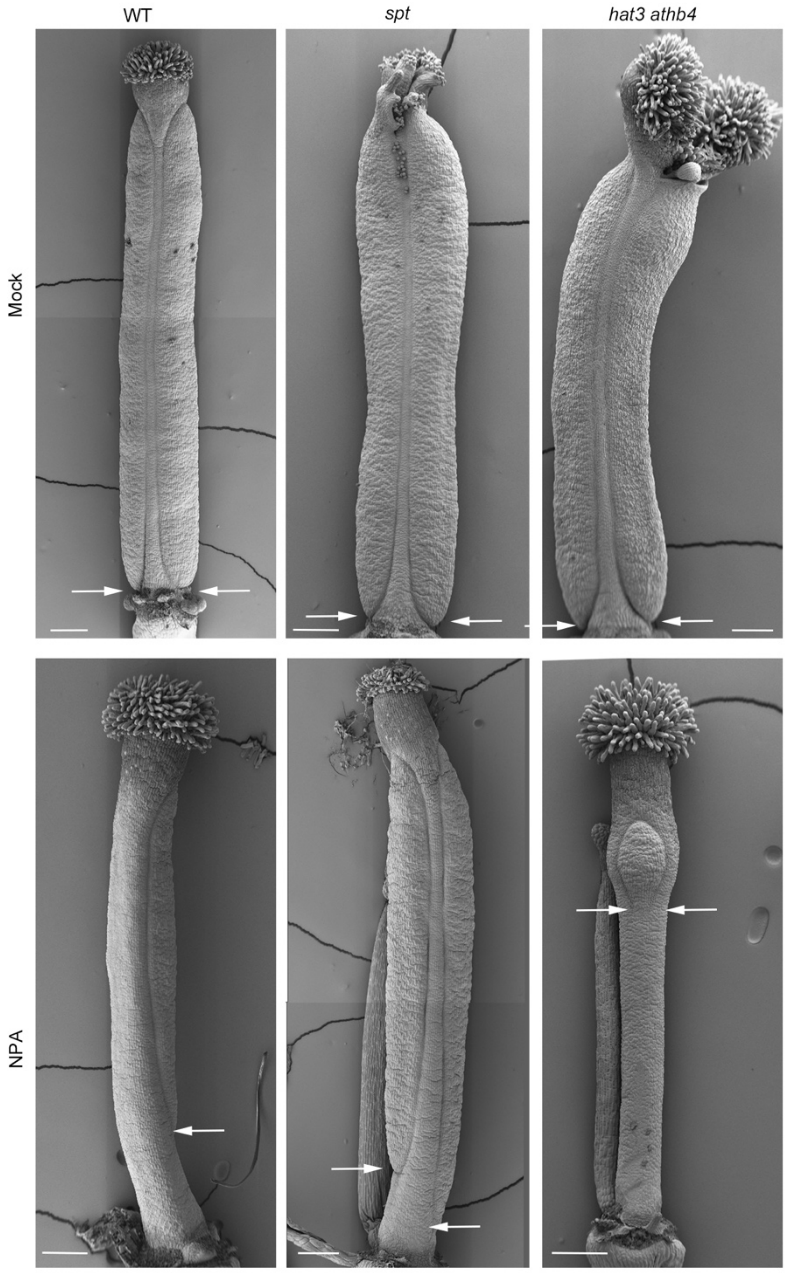

Supplementary Figure 7. *hat3 athb4* mutant gynoecium is hypersensitive to NPA treatment. SEM images of gynoecia at stage 13 of Col-0 wild-type (left), *spt* (centre), and *hat3 athb4* (right) treated with either Mock (top) or 100 µm of NPA (bottom). White arrows indicate the proximal end of each valve. Scale bars represent 200 µm. Similar results were obtained from two independent experiments.

Supplementary Figure 8

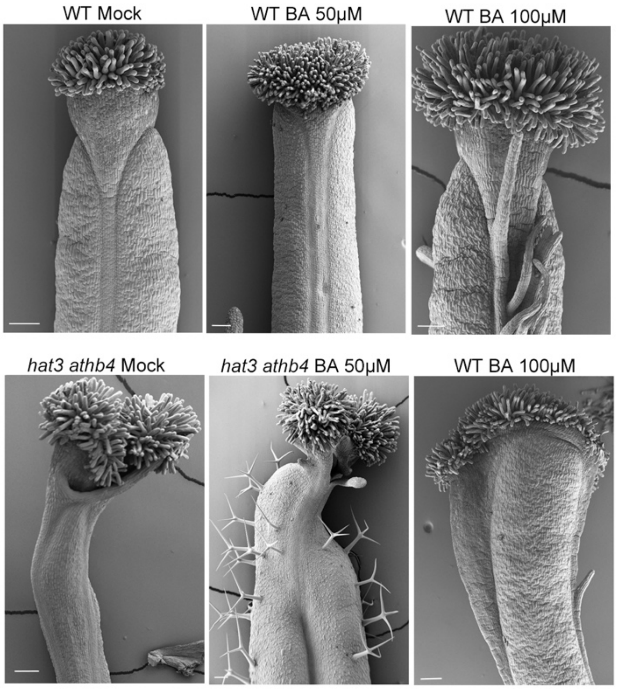

Supplementary Figure 8. HAT3 and ATHB4 suppress cytokinin sensitivity in the gynoecium. SEM images of Col-0 wild-type and *hat3 athb4* gynoecia treated either with Mock (left) or cytokinin (BA) 50 μM (centre) or 100 μM BA (right), as depicted on individual panels. Scale bars represent 100 μm. Similar results were obtained from two independent experiments.
